# Mitochondrial Dysfunction in Cardiovascular Diseases

**DOI:** 10.3390/ijms26051917

**Published:** 2025-02-23

**Authors:** Han-Mo Yang

**Affiliations:** Division of Cardiology, Department of Internal Medicine, Seoul National University Hospital, Seoul 03080, Republic of Korea; hanname@gmail.com; Tel.: +82-2-2072-4184

**Keywords:** mitochondrial dysfunction, cardiovascular disease, oxidative stress, mitochondrial dynamics

## Abstract

Mitochondrial dysfunction is increasingly recognized as a central contributor to the pathogenesis of cardiovascular diseases (CVDs), including heart failure, ischemic heart disease, hypertension, and cardiomyopathy. Mitochondria, known as the powerhouses of the cell, play a vital role in maintaining cardiac energy homeostasis, regulating reactive oxygen species (ROS) production and controlling cell death pathways. Dysregulated mitochondrial function results in impaired adenosine triphosphate (ATP) production, excessive ROS generation, and activation of apoptotic and necrotic pathways, collectively driving the progression of CVDs. This review provides a detailed examination of the molecular mechanisms underlying mitochondrial dysfunction in CVDs, including mutations in mitochondrial DNA (mtDNA), defects in oxidative phosphorylation (OXPHOS), and alterations in mitochondrial dynamics (fusion, fission, and mitophagy). Additionally, the role of mitochondrial dysfunction in specific cardiovascular conditions is explored, highlighting its impact on endothelial dysfunction, myocardial remodeling, and arrhythmias. Emerging therapeutic strategies targeting mitochondrial dysfunction, such as mitochondrial antioxidants, metabolic modulators, and gene therapy, are also discussed. By synthesizing recent advances in mitochondrial biology and cardiovascular research, this review aims to enhance understanding of the role of mitochondria in CVDs and identify potential therapeutic targets to improve cardiovascular outcomes.

## 1. Introduction

Cardiovascular diseases (CVDs) have been the leading cause of global morbidity and mortality [1]. Despite substantial advancements in treatment, the global burden of CVD continues to increase, underscoring the critical need for novel therapeutic targets. Mitochondria, often referred to as the powerhouses of the cell, are essential for maintaining cardiac and vascular function. They generate adenosine triphosphate (ATP) through oxidative phosphorylation, regulate calcium homeostasis, and modulate reactive oxygen species (ROS) production. Dysregulation of these processes plays a significant role in the development and progression of CVD [2,3].

Mitochondrial dysfunction is characterized by impaired ATP production, excessive ROS generation, and disrupted mitochondrial dynamics. These abnormalities compromise cellular homeostasis, resulting in cardiomyocyte apoptosis, endothelial dysfunction, and vascular remodeling [4,5]. For instance, in heart failure, mitochondrial dysfunction reduces ATP synthesis, impairing cardiac contractility and relaxation. Similarly, in ischemic heart disease, mitochondrial dysfunction exacerbates ischemia–reperfusion injury, leading to cell death and tissue damage [6,7].

The recognition of mitochondrial dysfunction as a key driver of CVD has created new opportunities for diagnosis and treatment. Emerging biomarkers of mitochondrial dysfunction, such as circulating mtDNA and oxidative stress markers, provide promising tools for early diagnosis and risk stratification [8,9]. Moreover, therapeutic strategies targeting mitochondrial pathways, including antioxidants, modulators of mitochondrial dynamics, and inhibitors of the mitochondrial permeability transition pore (mPTP), offer significant potential for improving cardiovascular outcomes [10,11].

This review aims to provide a comprehensive overview of the role of mitochondrial dysfunction in CVD, with a particular focus on its mechanisms, diagnostic biomarkers, and therapeutic interventions. By elucidating the molecular and cellular basis of mitochondrial dysfunction, this review seeks to identify novel strategies for the prevention and treatment of CVD.

## 2. Mechanisms of Mitochondrial Dysfunction in CVD

**Figure 1:**
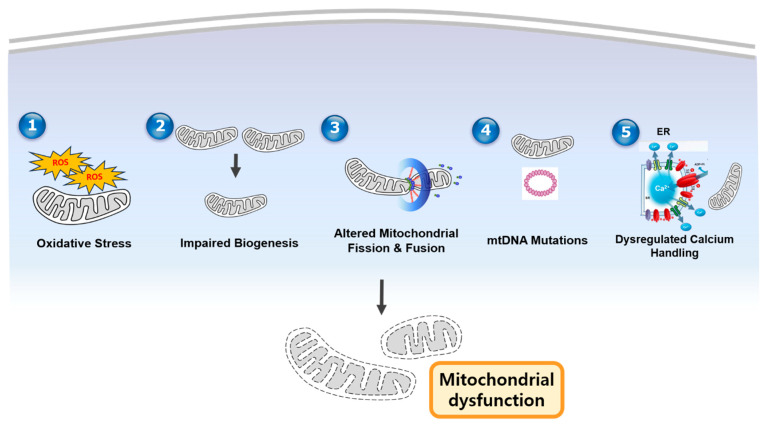
**Schematic figure of mechanisms of mitochondrial dysfunction in CVDs.** Oxidative stress (ROS), impaired mitochondrial biogenesis, altered mitochondrial dynamics (excessive fission and/or reduced fusion), mtDNA mutations, and dysregulated calcium handling collectively contribute to mitochondrial dysfunction, impairing ATP production and promoting cellular damage in cardiovascular diseases.

The following figure outlines the key mechanisms underlying mitochondrial dysfunction in CVD, including oxidative stress, impaired biogenesis, altered dynamics, mtDNA mutations, and calcium-handling defects (Figure 1).

Oxidative stress arises when the production of ROS surpasses the capacity of antioxidant defenses. Mitochondria are a primary source of ROS, generated as byproducts of oxidative phosphorylation. Under physiological conditions, low levels of ROS act as signaling molecules, regulating processes such as proliferation and apoptosis. However, excessive ROS production causes damage to proteins, lipids, and DNA, leading to cellular injury and inflammation [12].

In CVD, oxidative stress plays a pivotal role in pathogenesis, driving endothelial dysfunction, atherosclerosis, and myocardial injury. For example, ROS oxidize low-density lipoprotein (LDL), producing oxidized LDL (oxLDL), a key factor in atherosclerotic plaque formation. OxLDL accumulates in the arterial wall, triggering inflammation and plaque development [13].

Mitochondrial biogenesis is the process by which new mitochondria are formed to meet the energy demands of cells. This process is regulated by a network of transcription factors and coactivators, including peroxisome proliferator-activated receptor gamma coactivator 1-alpha (PGC-1α), nuclear respiratory factors (NRF-1/2), and mitochondrial transcription factor A (TFAM). PGC-1α serves as the master regulator of mitochondrial biogenesis, activating NRF-1/2 and TFAM to enhance the expression of mitochondrial genes [14,15].

Mitochondrial dynamics, encompassing fission and fusion, are critical for maintaining mitochondrial function and cellular homeostasis. Fission is mediated by dynamin-related protein 1 (Drp1), while fusion is regulated by mitofusins (Mfn1/2) and optic atrophy 1 (OPA1). A balance between fission and fusion ensures proper mitochondrial distribution, quality control, and energy supply [16,17].

In CVD, disruptions in mitochondrial dynamics often lead to mitochondrial fragmentation and dysfunction. Excessive fission, driven by Drp1 activation, results in the formation of small, dysfunctional mitochondria prone to ROS generation and apoptosis. For instance, in heart failure, heightened Drp1 activity contributes to cardiomyocyte apoptosis and disease progression. Conversely, impaired fusion decreases mitochondrial connectivity, disrupting energy distribution and cellular function [18,19].

mtDNA encodes 13 essential proteins of the electron transfer chain (ETC). Due to its proximity to ROS and lack of protective histones, mtDNA is particularly vulnerable to oxidative damage. Accumulation of mtDNA mutations disrupts ETC function, reducing ATP production and increasing ROS generation [20]. This vicious cycle exacerbates mitochondrial dysfunction and contributes to the progression of CVD [20].

In aging-related CVD, mtDNA mutations accumulate over time, impairing mitochondrial function and promoting oxidative stress. For instance, mutations in mtDNA-encoded Complex I and III genes are associated with cardiomyopathy and heart failure. Moreover, mtDNA damage releases mitochondrial damage-associated molecular patterns (DAMPs), which activate the innate immune response and promote inflammation and atherosclerosis [21,22].

Mitochondria play a crucial role in intracellular calcium regulation, which is essential for excitation–contraction coupling in cardiomyocytes. Calcium enters mitochondria through the mitochondrial calcium uniporter (MCU) and is extruded via the sodium–calcium exchanger (NCLX) [23]. Proper calcium handling is vital for maintaining mitochondrial function and cellular homeostasis [23].

In CVD, dysregulated calcium handling leads to mitochondrial calcium overload, triggering apoptosis and arrhythmias. For example, in heart failure, impaired calcium uptake and extrusion exacerbate mitochondrial dysfunction and disease progression. Additionally, calcium overload promotes the opening of the mPTP, resulting in cell death during ischemia–reperfusion injury [24,25] (Table 1).

Mitochondrial morphology is intricately linked to its function. In healthy cells, mitochondria exhibit a dynamic network with well-defined cristae, the inner membrane folds that house the ETC complexes. In CVD, however, significant morphological alterations are frequently observed. These include mitochondrial enlargement (swelling), fragmentation, and cristae remodeling [26].

In terms of causes of morphological alterations, several factors contribute to these changes [26]. Oxidative stress, as discussed previously, can directly damage mitochondrial membranes and proteins, leading to swelling and cristae disruption [5]. Imbalances in mitochondrial dynamics, particularly increased fission and reduced fusion, result in fragmented mitochondria [18,27,28]. Furthermore, defects in proteins responsible for cristae structure, such as OPA1 and the mitochondrial contact site and cristae-organizing system (MICOS) complex, can lead to abnormal cristae morphology [29]. Ischemia–reperfusion injury is a potent inducer of morphological changes due to rapid changes in ion concentrations and ROS production [30].

**Table 1:** Mechanisms of mitochondrial dysfunction in cardiovascular diseases.

Mechanism	Description	Associated CVDs	References
Impaired OXPHOS	Reduced ATP production due to defects in electron transport chain complexes	Heart failure, ischemic heart disease	[12,13,31,32,33,34,35]
mtDNA mutations	Accumulation of mutations leading to defective mitochondrial proteins	Cardiomyopathy, atherosclerosis	[20,21,22]
Excessive ROS production	Overproduction of ROS causing oxidative damage to lipids, proteins, and DNA	Hypertension, heart failure	[12,13]
Altered mitochondrial dynamics	Imbalance in fission/fusion and defective mitophagy	Myocardial infarction, hypertrophy	[16,17,18,19]
Calcium mishandling	Disrupted Ca^2+^ homeostasis leading to mitochondrial permeability transition	Arrhythmias, ischemia–reperfusion injury	[23,24,25]

OXPHOS, oxidative phosphorylation; mtDNA, mitochondrial DNA; ROS, reactive oxygen species.

Regarding consequences of morphological alterations, enlarged mitochondria often exhibit reduced respiratory capacity due to the dilution of matrix components and disruption of the ETC [31]. Cristae remodeling, characterized by the loss of cristae junctions and disorganization of the cristae structure, significantly impairs oxidative phosphorylation [32]. This is because the ETC complexes are optimally arranged within the cristae to facilitate efficient electron transfer. Disrupted cristae lead to electron leakage and increased ROS production [33]. Fragmented mitochondria, resulting from excessive fission, are also more prone to mitophagy, but if mitophagy is impaired, these dysfunctional mitochondria can accumulate and contribute to cellular damage [34]. Specific examples include studies showing that in heart failure, cardiomyocytes exhibit enlarged mitochondria with disorganized cristae, correlating with reduced ATP production [35]. Similarly, in ischemic heart disease, cristae remodeling is a key feature of mitochondrial damage following reperfusion [36]. Targeting the mechanisms that drive these morphological changes is a potential therapeutic strategy [37]. For example, modulating mitochondrial dynamics proteins or improving cristae structure through interventions that enhance OPA1 function could be beneficial [38].

Beyond energy production via the ETC and ATP synthesis, mitochondria serve as central hubs of cellular metabolism through the tricarboxylic acid (TCA) cycle [39]. The TCA cycle integrates carbohydrate, lipid, and amino acid metabolism, producing Nicotinamide Adenine Dinucleotide (NADH) and Flavian Adenine Dinucleotide (FADH2) to fuel the ETC while maintaining redox balance via NAD+/NADH ratios [40]. Pyruvate dehydrogenase (PDH), partially encoded by mtDNA, regulates carbon flux into the TCA cycle by converting pyruvate to acetyl-CoA [41]. In CVD, low PDH activity—often due to oxidative stress or mtDNA damage—leads to metabolic inflexibility, a hallmark of conditions like heart failure and diabetic cardiomyopathy [42]. This inflexibility shifts cardiac energy reliance from fatty acid oxidation to glycolysis, reducing ATP efficiency and exacerbating oxidative stress as NADH accumulates without adequate ETC processing [43]. For instance, in heart failure, reduced TCA cycle activity correlates with diminished ATP production and increased ROS [44]. Additionally, the TCA cycle’s interplay with the ETC is critical for redox homeostasis; disruptions in either amplify ROS production, creating a vicious cycle of mitochondrial dysfunction [45]. Therapeutic strategies, such as PDH activators (e.g., dichloroacetate) or metabolic modulators, aim to restore TCA cycle flux and redox balance, offering potential benefits in CVD management [46].

## 3. Mitochondrial Dysfunction in Specific Cardiovascular Diseases in Various Cell Types

Mitochondrial dysfunction plays a central role in the pathogenesis of various CVDs, including heart failure, ischemic heart disease, hypertension, and atherosclerosis [47]. This section examines the mechanisms through which mitochondrial dysfunction contributes to these conditions, focusing on molecular pathways and therapeutic implications.

In terms of energetic substrate preferences in cardiovascular cells, the cardiovascular system comprises diverse cell types—cardiomyocytes, endothelial cells, macrophage, and vascular smooth muscle cells (VSMCs)—each with distinct energetic demands and substrate preferences that shift in CVD [48]. In healthy cardiomyocytes, fatty acid oxidation (FAO) dominates, contributing 70% of ATP via the TCA cycle and ETC, with glucose oxidation as a secondary source [49]. This preference reflects the heart’s high energy demand and mitochondrial density [50]. Endothelial cells, conversely, rely primarily on glycolysis (85% of ATP), despite ample oxygen availability, preserving mitochondrial function for ROS signaling rather than energy production [51]. VSMCs also favor glycolysis under physiological conditions but can adapt to FAO during proliferation or stress [52]. Macrophages play a critical role in the inflammatory response in atherosclerosis. Their metabolic state is closely linked to their activation status [53]. Pro-inflammatory (M1) macrophages exhibit increased glycolysis and reduced oxidative phosphorylation, similar to the Warburg effect observed in cancer cells [54]. This metabolic shift supports the rapid production of inflammatory mediators [55]. Anti-inflammatory (M2) macrophages, on the other hand, rely more on oxidative phosphorylation and FAO [56]. In CVD, these preferences shift pathologically [57]. In heart failure, cardiomyocytes exhibit metabolic inflexibility, reducing FAO and increasing glycolysis, which lowers ATP yield and elevates ROS due to TCA cycle and ETC dysfunction [58,59,60]. Endothelial cells in hypertension or atherosclerosis increase mitochondrial FAO under oxidative stress, impairing NO production and exacerbating dysfunction [61]. VSMCs in hypertension shift toward glycolysis and FAO, promoting proliferation and vascular remodeling [62]. These shifts highlight cell-specific mitochondrial vulnerabilities and suggest targeted therapies, such as FAO enhancers (e.g., PPAR agonists) for cardiomyocytes or glycolysis modulators for endothelial cells, to restore metabolic balance in CVD [63].

Heart failure, characterized by impaired cardiac contractility and relaxation, results in reduced cardiac output and systemic perfusion. Mitochondrial dysfunction is a hallmark of heart failure, driving energy depletion, oxidative stress, and cardiomyocyte apoptosis [47]. The heart’s high energy demand depends on mitochondria for ATP generation via oxidative phosphorylation. In heart failure, impaired ATP production leads to energy starvation and contractility deficits, compounded by reduced mitochondrial biogenesis and downregulation of fatty acid oxidation [41]. Reduced ATP levels in failing hearts, compared to non-failing controls, have been observed in human studies [43]. Similarly, decreased expression of PGC-1α, a key regulator of mitochondrial biogenesis, correlates with impaired mitochondrial function in failing hearts [60].

Excessive ROS production is another defining feature of mitochondrial dysfunction in heart failure, leading to oxidative damage of cellular components. Elevated ROS disrupt calcium handling and excitation–contraction coupling, promoting arrhythmias and reduced contractility [45]. Animal models of heart failure have shown increased ROS levels, contributing to oxidative damage and cardiac dysfunction [46]. However, antioxidant defenses, such as superoxide dismutase (SOD) and glutathione peroxidase (GPx), are often overwhelmed, resulting in sustained oxidative stress. For instance, reduced SOD activity in failing human hearts has been associated with increased oxidative damage [64].

Mitochondrial dysfunction also promotes cardiomyocyte apoptosis through intrinsic pathways. Calcium overload and ROS trigger mPTP opening, leading to cytochrome c release and caspase activation, which exacerbate cell loss and disease progression [65]. Inhibition of mPTP opening has shown protective effects in preclinical heart failure models, reducing apoptosis and improving cardiac function [66]. Additionally, elevated caspase activity correlates with disease severity in human heart failure [67].

Ischemic heart disease, caused by restricted myocardial blood flow, leads to ischemia and infarction. Although reperfusion is essential to restore oxygen supply, it can exacerbate damage through ischemia–reperfusion injury (IRI). Mitochondrial dysfunction is central to IRI, with oxidative stress, calcium overload, and mPTP opening driving cell death [68]. During ischemia, oxygen deprivation reduces ATP production while increasing ROS generation. Reperfusion introduces a sudden influx of oxygen and calcium, aggravating mitochondrial damage and triggering mPTP opening [69]. Studies have shown that inhibiting mPTP reduces infarct size and improves cardiac function in experimental IRI models [70].

Calcium overload during IRI is a major contributor to mitochondrial dysfunction, directly inducing mPTP opening and apoptosis in cardiomyocytes [71]. Similarly, excessive ROS production during reperfusion overwhelms antioxidant defenses, causing oxidative damage that impairs the ETC and exacerbates cell death [72]. A dramatic increase in ROS levels during reperfusion has been shown to impair cardiac function [73].

Therapeutic strategies targeting ROS have demonstrated potential in mitigating IRI. For example, antioxidant therapies like N-acetylcysteine (NAC) and MitoQ reduce ROS production and improve cardiac outcomes. MitoQ has been shown to decrease infarct size and oxidative damage in preclinical IRI models [74].

Hypertension is characterized by persistently elevated blood pressure and vascular remodeling. Mitochondrial dysfunction in endothelial and vascular smooth muscle cells significantly contributes to its pathogenesis by inducing oxidative stress, inflammation, and vascular dysfunction [50]. Endothelial cells play a vital role in maintaining vascular tone and blood pressure, but mitochondrial dysfunction in these cells impairs nitric oxide (NO) production, leading to endothelial dysfunction and vasoconstriction. Excessive ROS generation in endothelial cells reduces NO bioavailability, hindering vasodilation and promoting hypertension [75]. Elevated ROS levels in hypertensive patients have been linked to endothelial dysfunction and reduced NO levels [76]. Antioxidant treatments, including vitamin C and Coenzyme Q10 (CoQ10), have shown efficacy in improving endothelial function and lowering blood pressure. For instance, vitamin C supplementation has been reported to enhance endothelial function in hypertensive patients [77].

In VSMCs, mitochondrial dysfunction exacerbates oxidative stress and inflammation, driving vascular remodeling. Increased ROS levels activate signaling pathways that lead to hypertrophy and fibrosis, resulting in vascular stiffness and elevated blood pressure [78]. Studies indicate that ROS production in smooth muscle cells is heightened in hypertension, contributing to these pathological changes [79]. Antioxidant therapies, such as tempol and apocynin, have demonstrated potential in mitigating vascular remodeling and improving blood pressure in both preclinical and clinical settings. For example, tempol has been shown to reduce vascular remodeling in experimental hypertension models [80].

Atherosclerosis, a chronic inflammatory disease, is characterized by plaque formation within the arterial wall. Mitochondrial dysfunction in endothelial cells and macrophages plays a pivotal role in its progression by inducing oxidative stress, inflammation, and foam cell formation [81]. OxLDL accumulation in arterial walls triggers inflammatory responses and promotes plaque development. ROS overproduction in macrophages further drives foam cell formation, a hallmark of atherosclerotic plaques [82]. Elevated ROS levels in atherosclerotic plaques have been linked to increased oxidative stress and inflammation [83]. Antioxidant therapies, such as statins and vitamin E, have shown promise in reducing these processes and improving vascular health. For example, statin therapy has been associated with decreased ROS production and improved endothelial function in patients with atherosclerosis [84].

Mitochondrial dysfunction also contributes to plaque instability and rupture by amplifying oxidative stress and inflammatory signaling. ROS generated in endothelial cells and macrophages enhances the expression of adhesion molecules and cytokines, facilitating immune cell recruitment and plaque growth. Furthermore, mitochondrial dysfunction in smooth muscle cells promotes instability and rupture of plaques, increasing the risk of acute cardiovascular events such as myocardial infarction and stroke [85]. Elevated ROS levels in unstable plaques have been identified as a major factor in plaque rupture, underscoring their role in disease progression [86]. Antioxidant strategies, including statins and vitamin E, have shown efficacy in stabilizing plaques and reducing rupture risk. For instance, vitamin E supplementation has been reported to decrease plaque instability in experimental models of atherosclerosis [87].

## 4. Diagnostic Biomarkers of Mitochondrial Dysfunction in CVD

Emerging biomarkers of mitochondrial dysfunction in CVDs include circulating mtDNA, oxidative stress markers, and imaging techniques. These biomarkers offer valuable insights into mitochondrial function and disease severity, enabling early diagnosis and risk stratification [88].

Circulating mtDNA is a promising indicator of mitochondrial dysfunction and cellular damage. Under normal conditions, mtDNA remains within mitochondria, shielded from degradation. However, during cellular stress or injury, mtDNA is released into circulation, signaling mitochondrial damage and inflammation [89]. This release occurs through mechanisms such as mitochondrial outer membrane permeabilization (MOMP), mPTP opening, or extracellular vesicle (EV) release. For instance, mPTP opening during ischemia–reperfusion injury facilitates the release of mtDNA into the cytosol and circulation, which triggers inflammation and tissue damage [69]. Studies have shown elevated circulating mtDNA levels in patients with various CVDs, including heart failure, ischemic heart disease, and atherosclerosis. For example, patients with acute myocardial infarction (AMI) exhibit higher circulating mtDNA levels compared to healthy individuals, with these elevated levels being associated with larger infarct sizes, worse cardiac function, and an increased risk of adverse outcomes [90].

Oxidative stress markers are another key indicator of mitochondrial dysfunction in CVD. As oxidative stress is a defining feature of mitochondrial dysfunction, measuring these markers in blood or tissues provides critical information about disease severity and progression [91]. Malondialdehyde (MDA), a byproduct of lipid peroxidation, is one such marker, with elevated levels observed in patients with heart failure. These elevated MDA levels correlate with disease severity and poorer outcomes [92]. Similarly, 8-hydroxy-2′-deoxyguanosine (8-OHdG), which reflects oxidative DNA damage caused by ROS, is elevated in the blood or urine of patients with coronary artery disease (CAD), predicting a higher risk of cardiovascular events [93]. Furthermore, reductions in antioxidant defenses, such as glutathione (GSH) and GPx activity, are indicative of mitochondrial dysfunction. Lower levels of GSH and GPx activity have been reported in hypertensive patients, where they are associated with endothelial dysfunction and vascular remodeling [94].

Imaging techniques provide non-invasive methods to assess mitochondrial function and structure, offering further insights into the role of mitochondrial dysfunction in CVD [95]. Positron emission tomography (PET) imaging using radiolabeled tracers, such as 11C-acetate, has been employed to evaluate mitochondrial metabolism and oxidative capacity in heart failure [96]. Reduced 11C-acetate uptake is indicative of impaired mitochondrial function and correlates with worse clinical outcomes [97]. Magnetic resonance imaging (MRI) techniques, including phosphorus-31 magnetic resonance spectroscopy (31P-MRS), have been used to measure mitochondrial energy metabolism by quantifying high-energy phosphate compounds like ATP and phosphocreatine (PCr). Patients with heart failure show reduced PCr/ATP ratios, which are linked to greater disease severity and adverse outcomes [98]. Additionally, fluorescence imaging with mitochondria-specific dyes, such as tetramethylrhodamine methyl ester (TMRM) and MitoTracker, allows real-time assessment of mitochondrial membrane potential and ROS production. These dyes have been used in animal models of ischemia–reperfusion injury to demonstrate mitochondrial depolarization and ROS overproduction [99].

Proteomic and metabolomic approaches provide a comprehensive understanding of mitochondrial function and dysfunction by analyzing mitochondrial protein expression and metabolite profiles [100]. Proteomic studies have identified dysregulated mitochondrial proteins associated with disease progression in heart failure, including alterations in ETC components and antioxidant enzymes [101]. Similarly, metabolomic analyses of key mitochondrial metabolites, such as ATP, NAD+, and acetyl-CoA, have revealed significant disruptions in energy metabolism [102]. These altered metabolite profiles in heart failure patients reflect impaired energy production and heightened oxidative stress [103].

## 5. Therapeutic Strategies Targeting Mitochondrial Dysfunction in CVD

Mitochondrial dysfunction is a central driver of CVDs, presenting a pivotal therapeutic target. Current and emerging strategies focus on pharmacological interventions, lifestyle modifications, and advanced gene or cell-based therapies.

Pharmacological strategies aim to restore mitochondrial function, mitigate oxidative stress, and enhance cellular energetics. Antioxidants such as CoQ10, MitoQ, and NAC have shown promising results. CoQ10, an essential ETC component, enhances ATP production and reduces ROS levels, with clinical trials demonstrating reduced mortality in heart failure patients [104]. MitoQ, a mitochondria-targeted antioxidant, has shown preclinical efficacy in ischemia–reperfusion injury and heart failure, with ongoing human trials exploring its potential [105]. NAC, a glutathione precursor, improves antioxidant defenses and endothelial function, particularly in hypertension and coronary artery disease [106].

Imbalanced mitochondrial dynamics—excessive fission or impaired fusion—contribute significantly to CVD pathology. Inhibitors of Drp1, such as Mdivi-1, reduce mitochondrial fragmentation and improve cardiac function in experimental heart failure models [107]. Likewise, activating Mfn2 promotes mitochondrial fusion, mitigates oxidative stress, and enhances cardiac performance in preclinical studies [108].

The mPTP is a key mediator of cell death in CVD. Cyclosporine A inhibits mPTP opening, reducing infarct size in acute myocardial infarction, though systemic side effects limit its use [109]. TRO40303, a novel inhibitor, has shown efficacy in reducing infarct size in preclinical models, with clinical trials in progress [110].

Lifestyle interventions, including exercise and dietary changes, play an essential role in improving mitochondrial health. Aerobic exercise enhances mitochondrial biogenesis and energy metabolism, while resistance training improves mitochondrial function, exercise capacity, and quality of life in heart failure patients [111,112]. Dietary approaches, such as caloric restriction and adherence to a Mediterranean diet, enhance mitochondrial efficiency, reduce oxidative stress, and lower CVD risk [113,114].

In addition to these pharmacological and lifestyle approaches, the field is witnessing exciting advancements in therapies that directly address the genetic and cellular underpinnings of mitochondrial dysfunction [115]. Gene therapy, for instance, offers the potential to correct underlying genetic defects [116]. One strategy involves nuclear gene therapy, delivering functional copies of nuclear-encoded mitochondrial genes using viral vectors, often adeno-associated viruses (AAVs) [117]. Preclinical studies targeting PGC-1α, a master regulator of mitochondrial biogenesis, have demonstrated improved mitochondrial and cardiac function in heart failure models [118,119]. A more direct, but challenging, approach is mitochondrial gene therapy, aiming to modify the mitochondrial genome itself [120]. Advances in gene editing technologies, such as MitoTALENs and CRISPR/Cas9, have enabled targeted elimination or repair of mutant mtDNA, with promising results in cell and animal models demonstrating a reduction in mtDNA mutation load [121]. Another strategy, allotopic expression, circumvents the challenges of direct mtDNA manipulation by expressing mitochondrial genes from the nucleus and developing mechanisms to target the resulting proteins back to the mitochondria [122].

Beyond gene therapy, mitochondrial transplantation represents a novel therapeutic avenue [123]. This technique involves the transfer of healthy mitochondria, either autologous or allogeneic, into damaged cells [124]. Various methods, including direct injection, co-incubation, and the use of cell-penetrating peptides, facilitate this transfer [125]. Preclinical studies, particularly in models of ischemia–reperfusion injury and heart failure, have shown that mitochondrial transplantation can lead to improved cardiac function and reduced infarct size, and early-phase clinical trials are currently exploring its feasibility and safety [126,127]. Furthermore, cell-based therapies, utilizing stem cell-derived cardiomyocytes or cardiac progenitor cells, offer another strategy [128]. Transplanting these cells, which contain healthy mitochondria, provides not only a source of functional mitochondria but also the potential for cell replacement and beneficial paracrine effects that stimulate endogenous repair mechanisms [129]. However, it is important to acknowledge that, despite the significant promise of these advanced therapies, challenges remain [130]. Efficient and targeted delivery of therapeutic agents to mitochondria, ensuring long-term efficacy, and mitigating potential immune responses are critical areas requiring further investigation to optimize these approaches and successfully translate them into clinical practice [131,132] (Table 2).

## 6. Discussion

Mitochondrial dysfunction has emerged as a critical factor in the pathogenesis of a wide spectrum of CVDs, impacting energy production, redox balance, calcium homeostasis, and cell survival. This review has synthesized current understanding of the mechanisms underlying mitochondrial dysfunction in CVD, explored diagnostic biomarkers, and examined a range of therapeutic strategies, from established pharmacological interventions to cutting-edge gene and cell-based therapies. However, translating this rapidly expanding knowledge into tangible clinical benefits requires a multi-faceted approach that embraces technological innovation, personalized medicine, and a critical awareness of translational challenges.

The field of mitochondrial research is undergoing a revolution driven by powerful new technologies [143]. Single-cell omics, including single-cell RNA sequencing (scRNA-seq), single-cell proteomics, and single-cell metabolomics, are providing unprecedented insights into the heterogeneity of mitochondrial functions within different cell types of the cardiovascular system [144]. These technologies have revealed, for example, that cardiomyocytes, endothelial cells, vascular smooth muscle cells, and infiltrating immune cells exhibit distinct mitochondrial gene expression profiles and metabolic adaptations in response to pathological stress in conditions like heart failure, atherosclerosis, and hypertension [145,146]. This cell-specific understanding is crucial for developing targeted therapies that address the unique mitochondrial defects in each cell type.

Beyond transcriptomics, advances in super-resolution microscopy, such as stimulated emission depletion (STED) microscopy and photoactivated localization microscopy (PALM), are enabling visualization of mitochondrial structure and dynamics at the nanoscale [147]. These techniques have revealed intricate details of cristae remodeling, mitochondrial network organization, and interactions between mitochondria and other organelles, providing insights into how these structural alterations contribute to CVD progression [148]. Furthermore, live-cell imaging techniques, including Förster resonance energy transfer (FRET)-based sensors, allow real-time monitoring of mitochondrial membrane potential, ROS production, and calcium dynamics, providing a dynamic view of mitochondrial function in living cells under physiological and pathological conditions [149].

Personalized approaches are increasingly recognized as vital for optimizing mitochondria-targeted therapies. Genetic testing can identify mutations in mitochondrial genes, such as PGC-1α and TFAM, which are linked to cardiomyopathies and heart failure [150]. Biomarker profiling of circulating mtDNA and oxidative stress markers further aids in stratifying patients based on therapeutic responsiveness [151]. Multi-omics approaches integrating proteomics, metabolomics, and transcriptomics provide comprehensive insights into mitochondrial dysfunction in CVD patients [152].

Precision therapies targeting specific mitochondrial dysfunctions are on the rise. For instance, pharmacogenomic analyses have identified patients with mitochondrial gene variants who respond more favorably to CoQ10 supplementation [153]. Novel interventions targeting mitochondrial dynamics, such as Drp1 inhibitors and Mfn2 activators, are showing promise in reducing oxidative stress and enhancing cardiac function in preclinical models [140].

Despite the exciting progress in mitochondrial research, significant challenges remain in translating preclinical findings into effective clinical therapies. One major hurdle is the lack of target specificity of many existing mitochondria-targeted drugs [132,143]. For instance, while antioxidants have shown promise in preclinical models, their clinical efficacy has been inconsistent, possibly due to off-target effects and interference with essential ROS signaling pathways [154]. Similarly, mPTP inhibitors can have unintended consequences on other cellular processes [109,110,155]. Developing more specific and targeted therapies that selectively modulate mitochondrial function without disrupting other cellular functions is a critical priority.

Another challenge is the efficient delivery of therapeutic agents to mitochondria within target cells. This is particularly relevant for gene therapies and mitochondrial transplantation, where the therapeutic payload needs to reach the mitochondria to exert its effect. Improving delivery methods, such as utilizing targeted nanoparticles, cell-penetrating peptides, or modified viral vectors, is an active area of research [156].

Species differences in mitochondrial biology and metabolism can also complicate the translation of preclinical findings to humans [157]. Animal models, while valuable, may not fully recapitulate the complexity of human CVD. Therefore, validating preclinical results in human cells and tissues, and ultimately in well-designed clinical trials, is essential [158].

## 7. Conclusions

Mitochondrial dysfunction plays a pivotal role in CVD by impairing energy production, increasing oxidative stress, and promoting cellular damage. Advances in mitochondrial research have identified promising diagnostic biomarkers and therapeutic targets, offering new opportunities to improve cardiovascular outcomes. Future efforts should prioritize uncovering molecular pathways of mitochondrial dysfunction, developing novel treatments, and addressing translational challenges. By focusing on these areas, the path toward personalized diagnosis and therapy for CVD can be paved, ultimately enhancing patient care worldwide.

## Figures and Tables

**Table 2:** Therapeutic strategies targeting mitochondrial dysfunction in CVDs.

Therapeutic Approach	Mechanism of Action	Clinical Applications	References
Mitochondrial antioxidants	Scavenge ROS and reduce oxidative stress	Heart failure, ischemic heart disease	[133,134]
Metabolic modulators	Shift metabolism from glycolysis to OXPHOS	Pulmonary hypertension, heart failure	[135,136]
Gene therapy	Correct mtDNA mutations or enhance mitochondrial biogenesis	Cardiomyopathy, ischemic heart disease	[137,138]
Mitochondrial fission/fusion modulators	Restore balance in mitochondrial dynamics	Myocardial infarction, hypertrophy	[139,140]
Mitophagy enhancers	Promote clearance of damaged mitochondria	Ischemia–reperfusion injury	[141,142]

OXPHOS, oxidative phosphorylation; mtDNA, mitochondrial DNA.

## Abbreviations

CVD	Cardiovascular Disease
ROS	Reactive Oxygen Species
ATP	Adenosine Triphosphate
mtDNA	mitochondrial DNA
OXPHOS	Oxydative Phosphorylation
mPTP	mitochondrial Permeability Transition Pore
LDL	Low Density Lipoprotein
PGC-1α	Peroxisome proliferator-activated receptor Gamma Coactivator 1-alpha
NRF-1/2	Nuclear Respiratory Factors–1/2
TFAM	Transcription factor A, mitochondrial
Drp1	Dynamin-related protein 1
Mfn1/2	Mitofusin 1/2
OPA1	Optic Atrophy 1
ETC	Electron Transport Chain
DAMPs	Damage-Associated Molecular Patterns
MCU	Mitochondrial Calcium Uniporter
NCLX	Sodium–Calcium Exchanger
MICOS	Mitochondrial contact site and Cristae-Organizing System
TCA	Tricarboxylic acid
NADH	Nicotinamide Adenine Dinucleotide
FADH2	Flavian Adenine Dinucleotide
VSMCs	Vascular Smooth Muscle Cells
FAO	Fatty Acid Oxidation
PPAR	Peroxisome Proliferator-Activated Receptor
SOD	Superoxide Dismutase
GPx	Glutathione Peroxidase
IRI	Ischemia–Reperfusion Injury
NAC	N-acetylcysteine
NO	Nitric Oxide
CoQ10	Coenzyme Q10
MOMP	Mitochondrial Outer Membrane Permeabilization
EV	Extracellular Vesicle
AMI	Acute Myocardial Infarction
MDA	Malondialdehyde
CAD	Coronary Artery Disease
GSH	Glutahione
PET	Positron Emission Tomography
MRI	Magnetic Resonance Imaging
PCr	Phosphocreatine
TMRM	Tetramethylrhodamine Methyl Ester
AAV	Adeno-Associated Viruses
MitoTALENs	Mitochondrially targeted Transcription Activator-like Effector Nucleases
CRISPR/Cas9	Clustered Regularly Interspaced Short Palindromic Repeats/CRISPR-Associated Protein 9
scRNA-seq	single-cell RNA sequencing
STED	Stimulated Emission Depletion
PALM	Photoactivated Localization Microscopy
FRET	Förster Resonance Energy Transfer
